# On the Dangers Arising from Syphilis in the Practice of Dentistry

**Published:** 1890-11

**Authors:** L. Duncan Bulkley


AMERICAN JOURNAL

—OF—

DENTAL SCIENCE.

VOL. XXIV. BALTIMORE, NOVEMBER, 1890. NO. 7.

ARTICLE I.

### ON THE DANGERS ARISING FROM SYPHILIS IN THE PRACTICE OF DENTISTRY.

BY L. DUNCAN BULKLEY, A. M., M. D.

(*Continued from page 250.*)

Roddick, ‡ of Montreal, has recorded a case of more than usual interest, where the primary syphilitic sore on the gum was undoubtedly the result of inoculation by means of dental forceps used in extracting a tooth; it is worth mentioning somewhat in detail. The patient was the wife of a physician, aged about thirty, and the mother of healthy children. She had always been in excellent health until about a year previous to her visit, when she had a tooth extracted, the operation being difficult and accompanied by considerable laceration of the gum. The wound showed no tendency to heal, but became sloughy and indurated. Within a few weeks the glands beneath

‡ Roddick, "Montreal Med. Journal," August, 1888, p. 93.

the jaw were found to be enlarged, and shortly an erythematous rash covered the body and extremities, followed later by a papular and squamous eruption, and sore throat and alopecia were soon added, to complete the picture of constitutional syphilis. Careful investigation failed to reveal any other source of contagion than the dental operation; the husband was entirely free from disease, and Dr. Roddick, who is an exceptionally careful man, and thoroughly qualified to judge, concluded that "in all probability the instrument used by the dentist was made the vehicle of contagion by being brought in contact with a mucous patch in the mouth of a syphilitic person previously operated upon."

2. Dangers to the *dental operator* from exposure to the syphilitic poison.

In the second division of this portion of our subject, namely, the dangers from syphilis to the operator in dental procedures, the number of instances on record is fewer, but they are very striking and well authenticated.

The first instance discovered was that of a dentist who reported his own case. * The inoculation took place on the middle finger of the left hand, above the nail, which was followed by constitutional syphilis. He could not trace the infection to any particular patient, but there could be no doubt that the poison came from mucous patches in some one's mouth, which lodged in one of the little cracks which so commonly come about the root of the nail.

Bumstead, † when speaking of the digital inoculation of accoucheurs, says that he has "known dentists to suffer in the same manner."

Neumann ‡ knew of a dentist who tore his hand on a sharp tooth while operating on a syphilitic patient, which injury was followed by severe syphilis.

---

* "Boston Med. and Surg. Journ.," vol. lviii. p. 38.

† Bumstead, "Venereal Diseases," edition of 1879, p. 432.

‡ Neumann, "Allg. Wien. Med. Zeitung," 1884, p. 61.

Jonathan Hutchinson, § in his excellent little clinical work on Syphilis, gives a plate of a well-marked circular, indurated chancre on the pulp of the finger of a dentist, which had been produced by a scratch on a patient's tooth.

Dr. Otis has recently, in a personal communication to me, related the following case, which is of peculiar interest, as it illustrates a method of infection in dentists which has not been previously noted. Mr. C., a dentist in large practice, applied for treatment with a chancre on the lips, accompanied with a general syphilitic eruption; his wife also contracted a chancre on the lip from him, with subsequent general syphilis. The infection was traced to a patient whom he had operated on, who had in the mouth a suspicious sore, claimed to be a "canker sore," but found afterwards to be syphilitic. The dentist was in the habit of occasionally holding instruments between the lips while operating, and the poison was conveyed thus to his lips by an instrument infected from the patient.

Although the number of these recorded instances of syphilis communicated in dentistry, which I have been able to find, after a very careful study of the subject during several years past, is relatively small, it is yet quite sufficient to establish the fact that such infection does occasionally take place, and to place us on our guard against such accidents in future. Undoubtedly but few of the cases occurring have ever found their way into print, and it is possible that when special attention has been called to the subject many more of them will be recognized and reported.

Having now considered the clinical basis on which rest the grounds for believing that there are dangers arising from syphilis in the practice of dentistry, we will examine the modes in which this accident can arise, and then consider the means for preventing the occurrence of this sad event.

---

§ Hutchinson, "Syphilis," London, 1887, plate ii., Fig. 2, p. 96.

Two methods of the non-venereal transmission of syphilitic virus are recognized, namely: First, the *direct*, and, second, the *indirect*.

1. *Direct syphilitic inoculation.*—In the first instance the poison is transferred directly from one individual to another, either through already existing wounds or in those occasioned at the time. The number of recorded cases of the communication of syphilis by kissing is now very great; hundreds can be found in literature, and I myself have seen over thirty cases of chancre of the lips. Infants at the breast of syphilitic nurses frequently acquire the disease, and breast-drawing by adults has given rise to numberless cases. In several series of instances syphilis has been both acquired and given by the application of the tongue to the eye to remove foreign particles and to heal disease; the poison has also been acquired and given in the process of wound-sucking, and other more rare modes of transmission which have been reported could be enumerated did time permit.

The particular method which is, perhaps, of most interest to us in the present connection is that of tooth wounds. The number of cases of this class which are on record is very great. Nearly all of them are from bites, usually intentional, and details of these need not be presented here. There are also a number where the infection has taken place from a blow on the mouth, the knuckles being wounded by the teeth. The first of these tooth-wound cases was observed near the beginning of the century by Boyer, * but not reported until 1840 by his son. Since that time a number of observers have recorded cases, some of whom have each seen several. Thus, Gamberini † saw three cases, C. Pellizzari ‡ three cases,

---

* Boyer, "Gaz. Med. de Paris;" Behrend's "Syphilidology," 1831, iii p. 322.

† Gamberini, "Gior. ital. d. mal. ven.," 1878, p. 365.

‡ Pellizzari, "Gior. ital. d. mal. ven.," 1882.

Van Harlingen § five cases, Lavergne and Perrin* five cases, Lesage † three cases, Finger ‡ four cases, and Jonathan Hutchinson § three or four cases.

In this connection may also be mentioned the fact that surgeons have repeatedly been inoculated in wounds occurring accidentally during operations on syphilitics, as also in other wounds when examining those with the disease.

When we consider the relatively large number of physicians and surgeons who have become thus accidentally inoculated in the discharge of professional duties (and I myself have seen at least seven or eight cases), the only wonder is that the accident does not occur oftener to dentists, whose fingers are continually bathed in the buccal secretions, often from mouths with active and intensely contagious syphilitic lesions.

Syphilis is rarely communicated to the patient in dental operations by the direct or immediate method of contact, although the cases of the communication of syphilis by tooth transplantation, already referred to, were probably by the direct method, the poison being undoubtedly carried directly in the transplanted tooth from a syphilitic to a healthy person, as the tooth was then inserted quickly after its removal.

It would also be possible for a dentist with an unrecognized chancre on the finger, in an early stage, to communicate the poison directly to a patient's mouth, as in the case of midwives, already mentioned; but no such instance has been found in literature.

2. *Indirect syphilitic inoculation.*—The second, indirect or mediate, method of contagion is that which

---

§ Van Harlingen, "Phil. Med. Times," 1884-85, xv. p. 80.

* Lavergne et Perrin, "Ann. de Derm. et de Syph.," 1884, 2d series, v. p. 332.

† Lesage, "Chancre par mosure," "These de Paris," 1885.

‡ Finger, "Die ven. Krankheiten," 1886, p. 14.

§ Jonathan Hutchinson, "Syphilis," London, 1887.

usually takes place in cases where the disease is transmitted during the operations of dentistry, and, indeed, in a very large proportion of the cases of syphilis innocently acquired.

Time and space would fail to tell of even a small share of the methods of mediate contagion of syphilis which are scattered throughout literature, but a brief statement of some of the principal modes by which it has been observed to be conveyed innocently from one person to another may aid us in understanding how the accident can occur in connection with dental operations.

As is well known, various household utensils, such as cups and drinking-vessels, spoons, tobacco-pipes, etc., have been the means of spreading the disease to hundreds of cases. The glass-blower's pipe has also caused a number of small epidemics, and cases have been traced to the assayer's blow-pipe, whistles, musical instruments, toys, pencils, pins, tack-nails, thread, paper-money, coin held in the mouth, etc. Of instruments used about the mouth, laryngo-scopes and tongue-depressors are peculiarly liable to transmit the disease, but the only instance found is a case reported by Jumon, * where a paper-cutter used to depress the tongue gave rise to syphilis. The cases traced to the use of the Eustachain catheter have been already mentioned.

Syphilis has also been conveyed by means of various fabrics, and a number of striking instances are on record where towels and napkins have transmitted the disease. An interesting case is given by Leloir, † where syphilis was communicated by means of a handkerchief.

Of peculiar interest are the cases on record, of which there are a number, where the disease has been transmitted by means of a tooth-brush. This was first observed by Blumenbach ‡ in the last century, and later by Baxter, §

* Jumon, "These de Paris," "Syph. ignoræ, 1830.
† Leloir, "Lecons sur la Syph.," Paris, 1886, p. 60.
‡ Blumenbach, "Bibliothek fur Aerzten," iii. p. 197.
§ Baxter, "Lancet," May 31, 1879.

and also by Bumstead and Taylor, * and more recently Haslund † has reported a similar case. Knight § has also recorded a case, seen by Bumstead, where a lady received a chancre of the tonsil apparently by means of tooth-powder, her syphilitic nephew dipping his brush into her box when cleaning his teeth.

It is understood, of course, in all these instances that the various objects served as a medium to convey the dried syphilitic secretion which adhered to them directly to the tissues of the individual who became infected, and it is readily seen how, unless precautions are exercised, the various implements and articles used in connection with dentistry may very easily become likewise the bearers of syphilitic virus.

It is not possible always to determine exactly upon which instrument, or in what manner, the poison is conveyed, but the preceding instances which have been cited show, on good authority, that the infection did take place in some way in connection with and in consequence of dental operations.

The agents and objects which may become the conveyors of the poison are as numerous as the implements and articles which may come in contact with the mouth in dental manipulations. For convenience these may perhaps be grouped in three or four classes, as follows: 1 instruments proper; 2, napkins; 3, rubber dams, wedges, dental floss, etc.; and, 4, plaster, rubber, etc., used in connection with the making of artificial teeth and sets.

Among instruments the only one plainly shown to have communicated the disease is the forceps, in the case related by Dr. Roddick, in which the site of the extracted tooth, where the gum was torn by the forceps, became a syphilitic ulcer, the seat of the chancre or primary sore of

---

* Bumstead and Taylor, "Path. and Treat of Ven. Dis.," Phila., 1879, p. 432.

† Haslund, "Monatshefte fur prakt. Dermat." 1885, p. 456.

§ Knight. "New York Med. Journ.," 1884, p. 662.

syphilis. But it is readily seen that the instruments used in excavating and plugging may also become infected, while there is peculiar danger in such instruments as files, burrs, and drills, where there are many depressions difficult of cleansing which may receive and retain the virus. Napkins and towels may convey the poison, as has been shown, while rubber dams, if used a second time, would very readily give infection from their prolonged contact with the soft parts. The same is true of wedges, a portion of which is often used for different patients, as also thread, ribbon, or dental floss passed between the teeth. The different workmen in a furrier's shop were once infected * from a thread passed between them and bitten off. My scanty knowledge of the process of taking casts and preparing and fitting artificial teeth does not permit me to speak in regard to any dangers arising from this, but I should judge that possibly accidental infection might occur in this line of work, perhaps quite as unexpectedly as it has happened in connection with other branches of dental practice.

We come, finally, to the most practical and important part of our subject,—namely, the prophylaxis, or prevention of the occurrence of this most unfortunate accident, the transmission of syphilis in the practice of dentistry. It may be somewhat out of place in the presence of this Society, including, as it does, many of the best elements of the dental profession in this city, to urge the simple matters of precaution about to be mentioned; but, as some may not heretofore have recognized the immense importance of the matter, it is best to err on the safe side, and to present briefly and clearly the precautions which seem to be indicated from what we know of the nature and virulence of the poison of syphilis.

As before mentioned, the virus or contagious element comes in liquid form from a chancre or a moist, exuding

* "Arch. f. Derm. u. Syph.," viii. 660.

surface, or, more rarely, with the blood itself, from a fresh wound. The secretion is very sticky and adherent, and when dried on an article forms a delicate coat, which could hardly be perceived. Nothing is known in regard to the viability of the virus, or the length of time during which it may retain its actively contagious character, but from many instances in the various conditions of life and in medical experience, it would seem that, under proper circumstances, it retains its vitality and activity for some considerable period longer than that possessed by the somewhat similar virus of vaccine. Days, weeks, or perhaps months after an instrument or article has become infected, it may again give off the poison and produce inoculation. Simple washing will not destroy the poison, although it may so dilute it that it becomes innocuous.

There are, however, several elements which are destructive to the life of the syphilitic poison, and these are heat and cold and the so-called disinfectants, antiseptics, or germicides. Cold can hardly be utilized practically, as a sufficiently low temperature for a requisite length of time would be difficult to maintain. Heat, however, may readily be employed in a thoroughly efficient manner, and is undoubtedly the very best means for destroying contagious principles. Inasmuch as dry heat, as in a flame, would injure instruments, as to their temper, the desired results are best obtained by means of moist heat, obtained in boiling water. Instruments and other articles placed in a vessel, and then subjected to vigorous boiling for half an hour or so, may be considered absolutely freed from any power of conveying contagious matter.

Various chemical substances are also capable of destroying the virus when efficiently used; prominent among these stand bichloride of mercury and carbolic acid. The mercurial salt has its disadvantages, both from its poisonous nature when in strong solution, and also from its corroding action on some metals. Carbolic acid, therefore, remains as the best of the two; and, indeed, when properly used, answers all the requirements of the case. But one must not be deceived by the odor, and be led to use a too weak solution, for it is questionable how serviceable the high dilutions used in anti-septic surgery are in overcoming such a poison as syphilis. The strong acid certainly destroys it, and a safe method is to dip the instruments in a ninety-five per-cent. solution, wiping or scrubbing them afterwards. A much weaker strength, possibly even a ten per-cent. solution, would probably be effective if they were left a considerable time in it and then thoroughly washed. I will not take your time in discussing other disinfectants, but only wish to impress the fact that not only for ordinary cleanliness, but also and particularly to avoid the possible danger of infection, too great care can hardly be spent in rendering instruments and everything pertaining to dental practice as absolutely clean as thought and labor can possibly make them.

Files and burrs are particularly liable to catch and hold the poison of syphilis in their fine serrations, and as they are also peculiarly liable to wound the soft parts they may really become means of contagion. Also the various articles connected with polishing the teeth are dangerous if not properly cared for. I well remember more than one dentist, in times past, polishing my own teeth with a bit of wood dipped in pumice-stone, which wood had evidently been used for former patients. Rubber dams and wedges can, of course, be readily disinfected in strong carbolic solutions, and napkins are rendered aseptic by boiling.

In regard to the personal prophylaxis of the dentist against acquiring syphilis in dentistry little need be said. The careful guarding of fresh wounds and thorough cleansing of the hands, and immediate sucking of any wound made during operations, will generally suffice to prevent the untoward event. "Forewarned, forearmed" applies well in regard to all the dangers from syphilis in dentistry. If the danger is thoroughly well known and appreciated, half the battle is won.

The question here arises, how far the dentist should be acquainted with syphilis, so as to be able to recognize it and avoid its dangers. Undoubtedly it would be most desirable that this knowledge should be obtained, but, unfortunately, a practical acquaintance with syphilis is somewhat difficult to obtain, except after considerable clinical experience in this branch of medicine. But it would certainly be extremely desirable that the mouth lesions should be known to dentists so as to be recognized, and this would be a fitting subject for the consideration of instructors in dental colleges.

A word may be added in regard to the duty of the physician in charge of syphilitic cases, when the patient may desire dental work to be performed. Should he prevent the patient from having the work done, and from thus exposing others, or should he acquaint the dentist with the diagnosis of the case and warn him against contaminating himself and others? The latter question involves an ethical point as to how far the medical adviser is right in revealing the nature of a patient's disease to others, and is a difficult one to answer; for professional relations require secrecy, especially in such a complaint. But, on the other hand, it cannot be right to wittingly expose others to the poison of syphilis. My practice has been to warn the patient earnestly of the danger, and, as far as in my power, to prevent his having dental work done while in the infective period, and especially while there were mouth lesions present, frequently examining the mouth for this purpose. If he failed to heed my instructions I should feel justified in warning his dentist.

In concluding our consideration of the topic of the evening, I beg to add that I have by no means wished to excite unnecessary alarm, but only to present facts already well known and conclusions which must be accepted by all those acquainted with the subject. With the presence of such a disease among us which often appears even in the best classes of society, it is well to recognize and understand the dangers which may arise therefrom, and to do our utmost to avert them. This, I trust, will be the result of the presentation of the subject which has been considered.—*International Dental Journal.*

